# Intermittent Fasting Alleviates Anesthesia/Surgery‐Induced Delirium‐Like Behavior in Aged Mice by Remodeling Gut Microbiota

**DOI:** 10.1002/cns.70748

**Published:** 2026-01-22

**Authors:** Peiying Huang, Longlu Cao, Tianyu Cao, Xueji Wang, Sichen Cui, Sufang Jiang, Huan Chen, Lichao Di, Sha Li, Lining Huang

**Affiliations:** ^1^ Postdoctoral Mobile Station The Second Hospital of Hebei Medical University Shijiazhuang Hebei China; ^2^ Department of Anesthesiology The Second Hospital of Hebei Medical University Shijiazhuang Hebei China; ^3^ Department of Anesthesiology Hebei General Hospital Shijiazhuang Hebei China; ^4^ Hebei Key Laboratory of Neurodegenerative Disease Mechanism Hebei Medical University Shijiazhuang Hebei China; ^5^ Department of Human Anatomy, Neuroscience Research Center Hebei Medical University Shijiazhuang Hebei China; ^6^ The Key Laboratory of Neural and Vascular Biology of Ministry of Education Shijiazhuang Hebei China; ^7^ Key Laboratory of Clinical Neurology (Hebei Medical University), Ministry of Education Shijiazhuang Hebei China

**Keywords:** gut microbiota, intermittent fasting, mitochondrial dynamics, postoperative delirium

## Abstract

**Background:**

Postoperative delirium (POD) is a serious complication in elderly patients, associated with prolonged recovery and adverse outcomes. Recent evidence links POD to mitochondrial dysfunction. While intermittent fasting (IF) has been shown to enhance mitochondrial function and exert neuroprotective effects, potentially through gut microbiota modulation, its ability to prevent POD and the underlying mechanisms remain unclear.

**Methods:**

We examined the effects of preoperative IF on delirium‐like behavior in aged mice following anesthesia/surgery. Assessments included neurobehavioral tests, gut microbiota composition, fecal shortchain fatty acids (SCFAs), hippocampal synaptic and mitochondrial ultrastructure via transmission electron microscopy, mitochondrial function, and related molecular markers. To establish causality, fecal microbiota transplantation and SCFA supplementation experiments were conducted.

**Results:**

Preoperative IF significantly attenuated anesthesia/surgery‐induced delirium‐like behaviors. Mechanistically, IF reshaped the gut microbiota and preserved SCFA levels, which collectively maintained hippocampal mitochondrial homeostasis. Both fecal microbiota transplantation and SCFA supplementation replicated the protective effects of IF, confirming the causal role of gut microbiota and its metabolites.

**Conclusion:**

These findings demonstrate that preoperative intermittent fasting mitigates delirium‐like behavior by modulating the gut microbiota–SCFA–mitochondrial axis, highlighting its potential as a non‐pharmacological strategy to enhance neurocognitive resilience and prevent POD in elderly surgical patients.

## Graphical abstract

Preoperative intermittent fasting alleviates postoperative delirium‐like behaviors in aged mice by remodeling gut microbiota and preserving shortchain fatty acids. This gut‐brain interaction maintains hippocampal mitochondrial dynamics and synaptic plasticity, offering a promising non‐pharmacological strategy for perioperative neuroprotection.

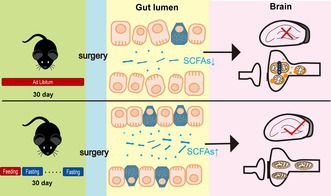

## 1 Introduction

Postoperative delirium (POD) is a prevalent complication among elderly patients, characterized by fluctuating changes in attention, cognition, and consciousness [1]. With projections indicating that by 2050, the population aged ≥ 65 will exceed 1.5 billion, the number of older adults undergoing anesthesia and surgical procedures is expected to increase significantly [2]. Emerging evidence highlights the critical role of gut microbiota in human health, with both animal studies and our clinical research demonstrating that perioperative gut microbiota dysbiosis constitutes a significant risk factor for POD [3, 4, 5, 6]. These findings suggest that optimizing perioperative gut microbiota may represent an effective preventive strategy for POD.

Diet is a powerful modulator of gut microbiota function and composition [7]. Recently Intermittent fasting (IF) stands out as a popular dietary restriction method. IF typically involves 16–48 h fasting periods with complete caloric abstinence (except water), distinguishing it from continuous caloric restriction [8, 9]. Current evidence indicates IF's benefits extend beyond reduced caloric intake, primarily deriving from metabolic switching between cellular energy states that enhances mitochondrial function [9, 10]. During fasting, mitochondria undergo fusion to increase energy storage capacity [11]. Notably, mitochondrial dysfunction has recently been linked to POD [12, 13], while intermittent bioenergetic challenges—including fasting, exercise, and cognitive training—enhance neuronal mitochondrial function and stress resilience, potentially reducing POD incidence [14, 15, 16, 17]. Further, IF may safeguard against cognitive impairment and synaptic plasticity deficits induced by sevoflurane anesthesia [18]. However, the precise mechanisms remain unclear, and whether gut microbiota mediates the protective effects against POD requires clarification.

Given the emerging reports that link gut microbiota, mitochondrial dysfunction, and postoperative neurocognition, we undertook a study to examine the impact of preoperative IF on anesthesia/surgery‐induced delirium‐like behavior and the underlying mechanisms in aged mice. Following anesthesia/surgery, we assessed neurobehavior, gut microbiota composition, fecal SCFAs, transmission electron microscopy of synaptic and mitochondrial morphology, mitochondrial function and relevant molecular markers in the hippocampus. To determine the role of the gut microbiota in mediating the protective effects of IF, we also conducted fecal microbiota transplantation experiments and SCFAs supplementation experiments. Our findings suggest that gut microbiota mediate the neuroprotective actions of preoperative IF on anesthesia/surgery‐induced delirium‐like behaviors, thus highlighting the potential of modulating the gut microbiota as an effective intervention for postoperative neurocognitive disorders.

## 2 Experimental Section

### 2.1 Animals: IF Model and Fecal Microbiota Transplantation

C57BL/6 female mice were purchased from Henan Sikebais Biotechnology Co. Ltd. (Anyang, China) and raised to 18 months of age. Only female mice were used in this study, as previous research indicates they are more susceptible to cognitive impairment following anesthesia or surgery [19]. All mice were housed in polypropylene cages at 22°C with a 12‐h light–dark cycle. Each cage accommodated five mice. Mice were fed a regular chow (SPF Rodent Maintenance feed, purchased from HuanYu BioTech. Ltd., China) and pure water. All procedures complied with protocols approved by the Institutional Animal Care and Use Committee at the Second Hospital of Hebei Medical University (2024‐AE285), and all data collection followed ARRIVE guidelines.

**FIGURE 1:**
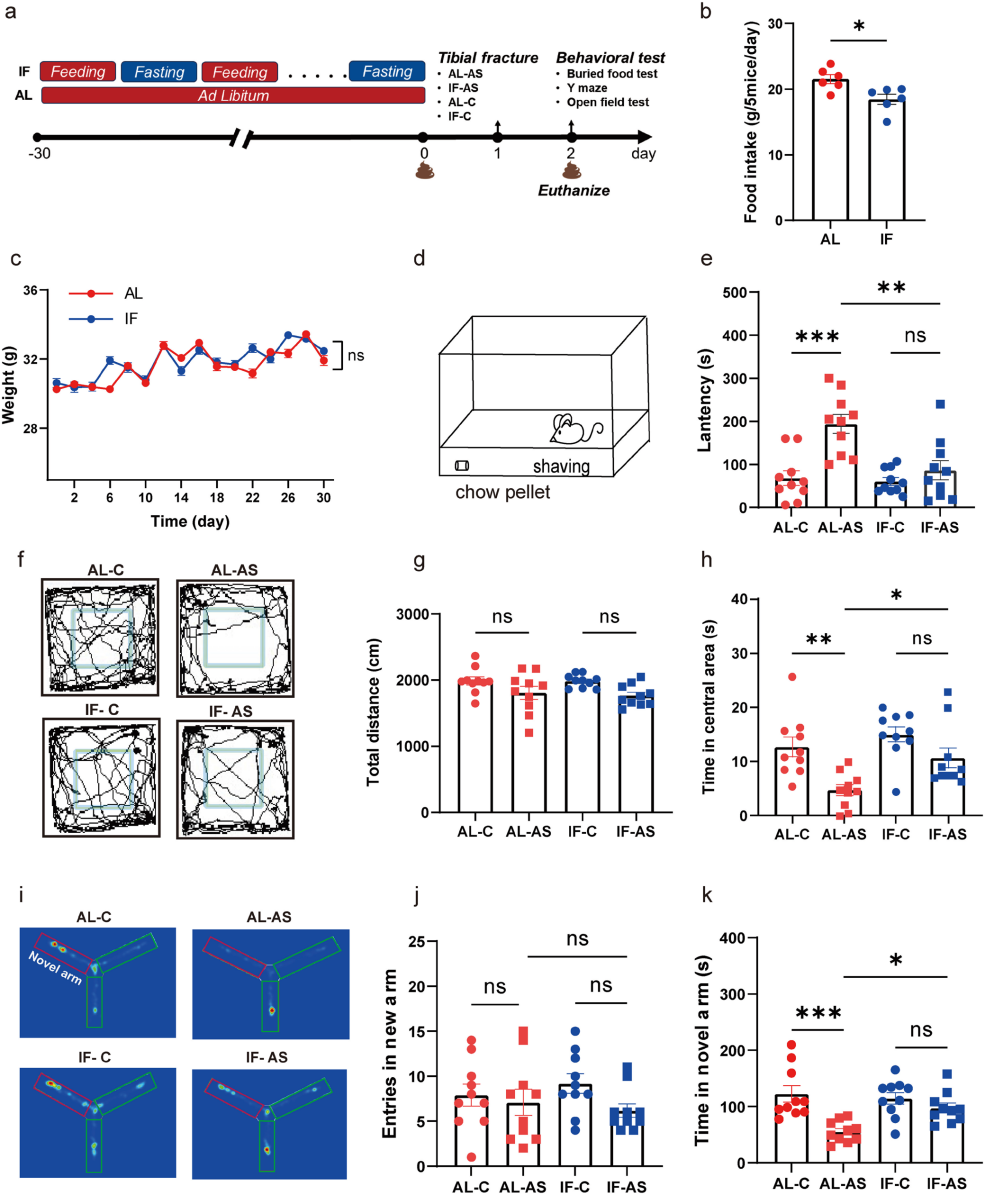
Intermittent fasting pre‐anesthesia/surgery alleviates behavioral deficits in a murine model of postoperative delirium. (a) Illustration of the experimental design. Mice in the intermittent fasting (IF) group underwent periodic feeding and fasting, while mice in the ad libitum (AL) group had free access to food. Following the 30‐day feeding period, mice were exposed to anesthesia/surgery (AS) or control (C) conditions. (b) Daily food intake of mice (*n* = 6 cages/group, 5 mice/cage). (c) Changes in mouse body weight over time (*n* = 10 mice/group). (d) Schematic representation of the buried food test. (e) Latency to locate food during the buried food test (*n* = 10 mice/group). (f) Typical paths traversed by mice during the open field assessment conducted 24 h post‐surgery (*n* = 10 mice/group). (g) Total distance traveled by mice in the open field. (h) Time spent in the central zone of the open field test. (i) Distinct navigation patterns of mice in the Y‐maze test, conducted 24 h post‐surgery (*n* = 10 mice/group). (j) Number of novel arm entries in the Y‐maze test. (k) Duration of exploration in the novel arm during the Y‐maze test. Results are presented as mean ± standard error of the mean (SEM). *p < 0.05; **p < 0.01; ***p < 0.001; ns, not significant.

For feeding and anesthesia/surgery experiments (Figure 1a), after 1 week of adaptation, mice were divided into an IF group and ad libitum (AL) group. Mice in the IF group were food‐deprived for 24 h every other day, with ad libitum access to food on intervening days, for a total of 30 preoperative days. They had free access to water, while the AL group had unrestricted access to both food and water. Body weight and food intake were recorded on fasting days, and fecal samples were collected pre‐surgery and post‐surgery and stored at −80°C. After 30 days, IF and AL groups were further divided into anesthesia/surgery groups (AL‐AS and IF‐AS) and control groups (AL‐C and IF‐C).

For fecal microbiota transplantation experiments (Figure 7a), mice were divided into F‐AL and F‐IF groups. Before transplantation, existing microbiota was depleted with a seven‐day course of broad‐spectrum antibiotics administered via oral gavage (100 μL of vancomycin, 5 mg/mL; metronidazole, 10 mg/mL) and mixed in drinking water (1 g/L ampicillin, 0.5 g/L neomycin) [20]. For transplantation, frozen stool samples were transferred to an anaerobic chamber, thawed, and homogenized to 125 mg/mL in reduced sterile PBS containing 20% glycerol. The filtered slurry was aliquoted and stored at −80°C. Mice were orally administered 0.1 mL of donor microbiota suspension every 2 days for 14 days. Following successful fecal microbiota transplantation, all groups underwent anesthesia/surgery, forming F‐AL + AS and F‐IF + AS groups.

For SCFAs supplementation experiments (Figure 9a), mice were divided into Con and SCFAs groups. For the SCFAs group, the SCFAs (acetate 67.5 mM, propionate 40 mM, butyric acid 25 mM) were dissolved in the drinking water of the mice [21]. The Con group received pure drinking water. After 14 days, all groups underwent anesthesia/surgery, forming Con+AS and SCFAs+AS groups.

### 2.2 Anesthesia/Surgery

We used a mouse model of tibial fracture to examine effects on the central nervous system and POD [22]. Briefly, mice were anesthetized with 3.0% sevoflurane and 50% oxygen at a flow rate of 3 L/min using a small animal anesthesia machine (RWD Life Science, San Diego, CA, USA). An incision was made on the left hind paw to access muscles, which were carefully dissociated. A 0.38‐mm stainless steel pin was inserted into the intramedullary canal of the tibia, followed by osteotomy, and the incision was sutured. A 2% lidocaine solution was applied locally before the incision, and 1% tetracaine hydrochloride mucilage was administered to the wound twice daily for pain management.

### 2.3 Behavioral Tests

We conducted a series of behavioral tests, including the buried food test, open field test, and Y‐maze test, to assess changes in natural and learned behaviors and examine the delirium‐like phenotype [23]. A video camera linked to Smart v3.0 animal tracking system software (Panlab, Barcelona, Spain) was used to monitor and analyze the activity of mice in the Y‐maze test and open field test.

#### 2.3.1 Buried Food Test

After acclimation, the testing mouse was placed into a clean cage with a sweetened cereal pellet buried in 3–5‐cm‐thick bedding. The latency of the mouse to eat the food was recorded. If the mouse failed to find the pellet within 5 min, the test ended.

#### 2.3.2 Open Field Test

A 40 × 40 cm box was placed in the enclosure, creating two areas: a center zone (20 × 20 cm) and the surrounding area. Mice were individually placed in the middle of the center zone, and parameters like time spent in the center zone and total distance were analyzed. The open field test lasted 5 min.

#### 2.3.3 Y Maze Test

The Y‐maze consisted of three arms positioned at 120° angles, with each arm measuring 34 cm in length, 8 cm in width, and 14 cm in height. Arms were randomly designated as the begin arm, old arm, and novel arm. In the first trial, mice were allowed to explore the begin and old arms, with the novel arm blocked. After 2 h, a second trial was conducted in which mice were placed in the same begin arm with free access to all three arms. Animal tracking software was used to monitor and analyze the time spent in the novel arm, total arm entries, and entries into the novel arm.

### 2.4 ATP Content, Separation of Hippocampal Mitochondria, and Mitochondrial Membrane Potential

ATP content in the hippocampal tissue was measured using an enhanced ATP assay kit (ATP‐2‐Y, Comin, Suzhou, China) following the manufacturer's protocol. Hippocampal mitochondria were isolated using a tissue mitochondrial extraction kit (C3606, Beyotime, Shanghai, China) in accordance with the manufacturer's instructions. Extraction was conducted to assess mitochondrial membrane potential and DRP1 protein levels. Mitochondrial membrane potential was evaluated using an enhanced mitochondrial membrane potential assay kit in combination with a JC‐1 staining kit (C2003S, Beyotime).

### 2.5 Intestinal Permeability Testing

The intestinal permeability assay method was performed according to previous literature [24]. Following the final behavioral assessment, mice were fasted for 4 h. They were then gavaged with FITC‐dextran (molecular weight: 4000; ST2940, Beyotime) at a dosage of 600 mg/kg. After 4 h, mice were euthanized to collect blood for serum extraction. A total of 50 μL of serum was diluted with 1 × PBS. Concentrations of FITC‐dextran were measured using an Infinite F200 PRO microplate reader (Tecan, Männedorf, Switzerland), with excitation at 485 nm and emission at 590 nm.

### 2.6 Western Blot

Hippocampal tissues were immediately collected, with a portion processed for extraction of hippocampal mitochondria and a portion frozen in liquid nitrogen for storage at −80°C. We followed a standard immunoblot analysis protocol [25]. We used primary antibodies against DRP1 (1:1000; 8570 T, Cell Signaling, Danvers, MA, USA), AMPKα (1:1000; 25,325, Cell Signaling), and p‐Thr172‐AMPK (1:1000; 2531S, Cell Signaling) and corresponding secondary antibodies conjugated to horseradish peroxidase (1:10,000; 511,203, ZenBio, Chengdu, China). Internal control for whole cell proteins was β‐actin (1:10,000; 205/136–1‐AP, Proteintech, Wuhan, China). Loading control for the mitochondrial fraction was mitochondrial protein VDAC1 (1:1000; 4866 T, Cell Signaling).

### 2.7 Transmission Electron Microscopy

Anesthetized mice were perfused with 4% paraformaldehyde and 2.5% glutaraldehyde, and 1 mm^3^ hippocampus blocks were collected. The slices were then postfixed in 2.5% glutaraldehyde (Servicebio, Wuhan, China) overnight at 4°C. The samples were embedded, sectioned, and stained with lead citrate and uranyl acetate using an ultramicrotome (UC7, Leica, Wetzlar, Germany). A transmission electron microscope (HT7700, Hitachi, Tokyo, Japan) was used to observe and image the thin slices.

### 2.8 Microbe Analysis

We used an Omega Biotek soil DNA kit (Norcross, GA, USA) to extract nucleic acids and amplified the bacterial 16S rDNA V3–V4 region via PCR (primers: F—AC TCCTACGGGAGGCAGCA, R—GGACTACHVGGGTWTCTAAT). To create our sequencing library, we assembled samples using the TruSeqNano DNA LT Library Prep Kit (Illumina, San Diego, CA, USA) and performed 2 × 250 bp double‐ended sequencing on an Illumina NovaSeq machine. We compared and annotated each ASV feature sequence against the Greengenes database and ensured raw data quality using the DADA2 approach, as detailed by QIIME 2 (https://docs.qiime2.org/2019.4/tutorials/overview/) and R packages (R version 3.6) The above steps were performed by Nanjing Paisenuo Gene Technology Co. Ltd. (Nanjing, China). Then, using the GENESCLOUD (https://www.genescloud.cn/home) online analysis tool, the α‐diversity, β‐diversity, and composition of intestinal flora were analyzed. Alpha diversity was applied to analyze the complexity of species diversity for the samples using the Pielou_e and Shannon indices. Beta diversity analysis (based on weighted_unifrac) was used to evaluate differences in the samples in terms of species complexity. Additionally, linear discriminant analysis (LDA) was performed to evaluate the impact of significant species (LDA score) by setting the LDA score at 3.5 and obtained the biomarkers in different groups.

### 2.9 Gas Chromatography–Mass Spectrometry

The fecal sample (100 mg) was homogenized with PBS in a ratio of 1 mg of fecal sample to 5 μL of PBS. The mixture was vortexed at 3000 rpm for 3 min and then centrifuged at 15,000 rpm and 4°C for 5 min. Fifty microliters of the supernatant were collected and mixed with 10 μL of 15% aqueous phosphoric acid solution. This was followed by the addition of 980 μL of methyl tert‐butyl ether (MTBE) and 10 μL of internal standard solution (4‐methylpentanoic acid) at an initial concentration of 1 mg/mL. The mixture was then vortexed at 3000 rpm for 3 min and centrifuged at 15,000 rpm and 4°C for 10 min. The supernatant was collected for analysis. For the gas chromatography–mass spectrometry (GC–MS) analysis, an Agilent 7890B‐7000C GC–MS system with a DB‐FATWAXUI column (0.25 mm ID × 0.25 μm film thickness, 30 m length) was used. The carrier gas was helium at a flow rate of 1.5 mL/min. An injection volume of 1 μL was used. The temperature program started at 60°C and increased at 20°C/min to 120°C. The temperature then increased at 5°C/min to 150°C and further increased at 25°C/min to 200°C, which was held for 3 min. The ionization source of mass spectrometry is electron ionization (EI), and the detection modes are multiple reaction monitoring (MRM) and selected ion monitoring (SIM). The injection was conducted in a splitless mode with a split ratio of 2:1. The ion source temperature was set at 230°C. The total run time for the analysis was 14 min.

### 2.10 Statistical Analysis

Data are presented as mean ± standard error of the mean (SEM). Body weight data between two groups were analyzed using two‐way repeated measures analysis of variance (ANOVA). Paired *t*‐tests were used to compare preoperative and postoperative shortchain fatty acid levels, while independent sample *t*‐tests were applied for other two‐group comparisons. For multiple‐group comparisons, two‐way ANOVA followed by Tukey's multiple comparisons test was performed. All statistical analyses were conducted using GraphPad Prism (version 9.5), with *p* < 0.05 considered statistically significant.

## 3 Results

### 3.1 IF Alleviated Anesthesia/Surgery‐Induced Delirium‐Like Behavior in Aged Mice

Throughout the 30‐day feeding phase (Figure 1a), mice in the IF group consumed significantly less food (13.79%) than the AL group (Figure 1b) but did not exhibit any significant changes in body weight (Figure 1c).

Delirium‐like behavior was assessed 24 h after surgery with a series of behavioral tests, including the buried food test, open field test, and Y‐maze test. Compared to the AL‐C group, the AL‐AS group had significantly increased latency in finding food in the buried food test (Figure 1d,e) and significantly decreased time spent in the central area of the open field test (Figure 1f–h) and in the new arm of the Y‐maze test (Figure 1i–k). Notably, preoperative IF restored these metrics to baseline levels, suggesting that preoperative IF can alleviate delirium‐like behaviors in aged mice following surgery.

**FIGURE 2:**
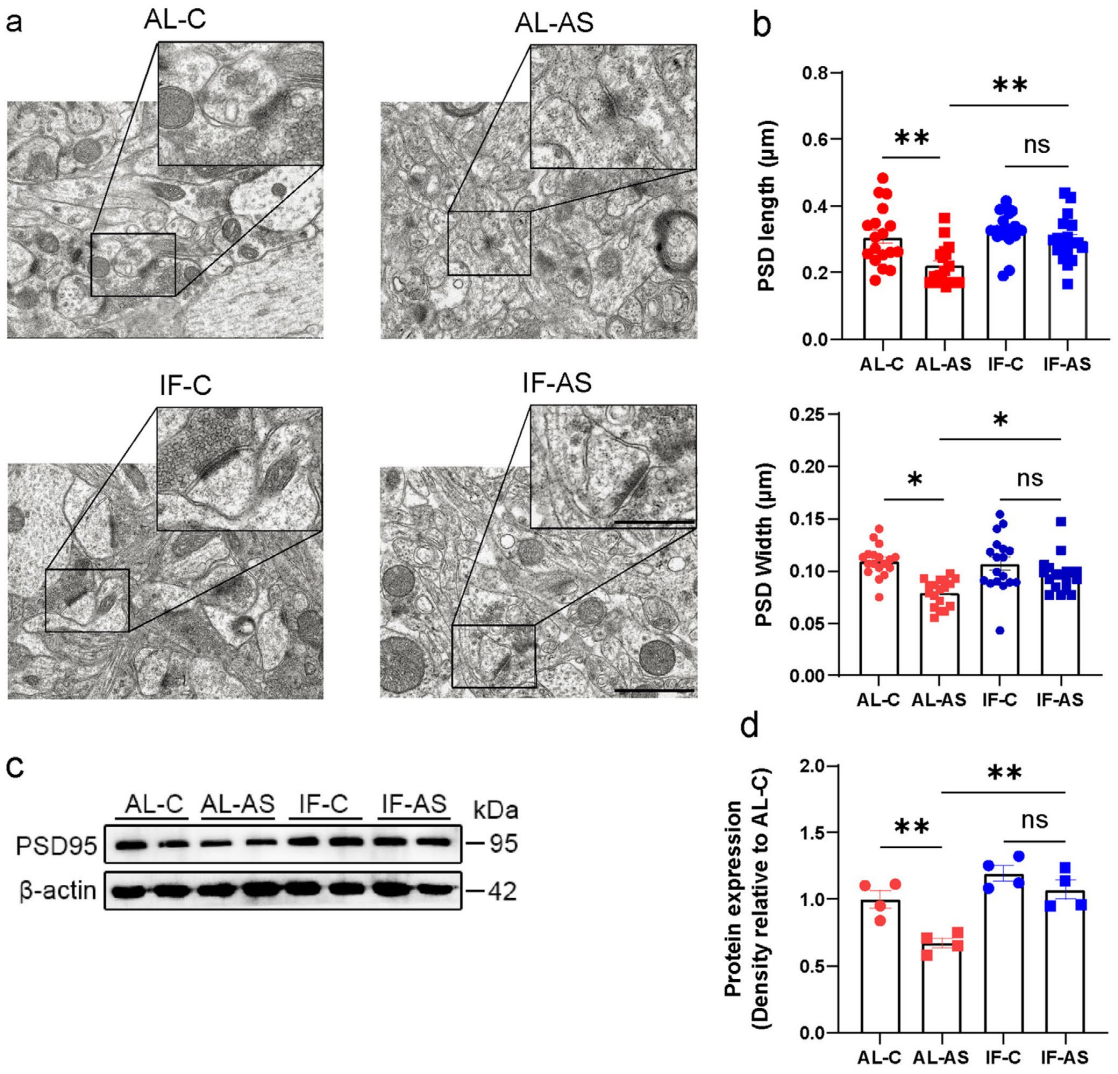
Intermittent fasting pre‐anesthesia/surgery alleviates damage to synaptic ultrastructure. (a) Representative transmission electron microscopy images of synaptic ultrastructure in mice in ad libitum (AL) and intermittent fasting (IF) groups, exposed to anesthesia/surgery (AS) or control (C) conditions. Scale bar = 1.0 μm (b) Measurements of postsynaptic density (PSD) length and width (*n* = 5 slices/group). (c, d) Representative western blot images and quantification of PSD95 levels in the hippocampus (*n* = 4 mice/group). Results are presented as mean ± standard error of the mean (SEM). *p < 0.05; **p < 0.01; ns, not significant.

Ultrastructural evaluation of synapses in the hippocampus—an essential area for cognition and memory—indicated that mice in the IF‐AS group had significantly increased postsynaptic density length and width compared to the AL‐AS group (Figure 2a,b). Accordingly, the hippocampus of mice in the IF‐AS group had significantly increased levels of PSD‐95, a crucial scaffolding protein in excitatory postsynaptic density, compared to the AL‐AS group (Figure 2c,d).

### 3.2 IF Reduced Anesthesia/Surgery‐Induced Mitochondrial Dynamics Disorders in the Hippocampus

**FIGURE 3:**
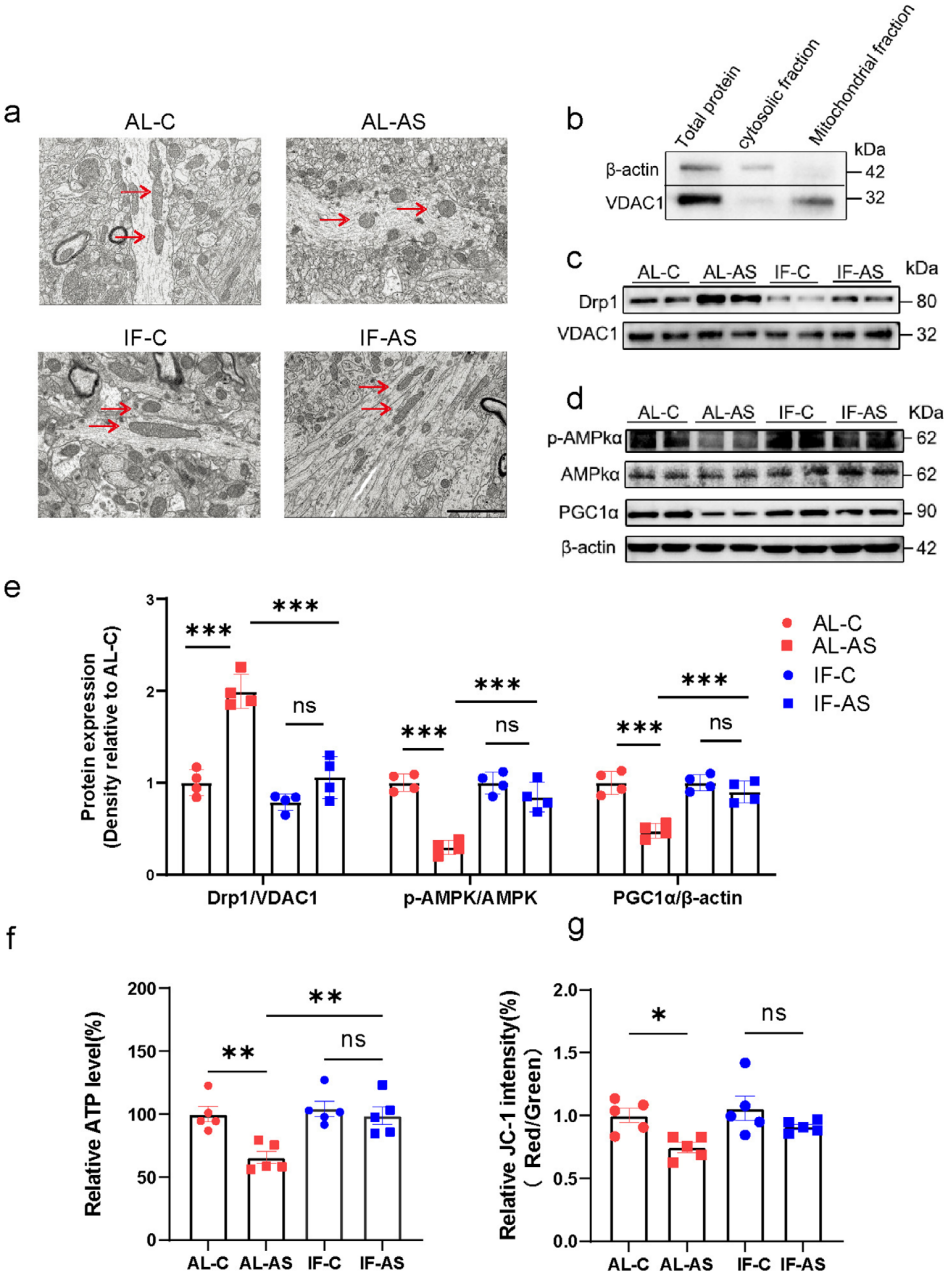
Intermittent fasting protects against anesthesia/surgery‐induced mitochondrial fission and the reduction of ATP production in the hippocampus. (a) Transmission electron microscopy images of mitochondrial morphology in neurons of mice in ad libitum (AL) and intermittent fasting (IF) groups, exposed to anesthesia/surgery (AS) or control (C) conditions. Scale bar = 2.0 μm. Red arrows indicate mitochondria. (b) Representative western blot images of β‐Actin and VDAC expression in total protein, cytosolic fraction, and mitochondrial fraction of mice. (c) Representative western blot images of DRP1 levels in hippocampal mitochondrial fractions of mice (*n* = 4 mice/group). (d) Representative western blot images of AMPK/PGC1α signaling pathway in the hippocampus of mice. (e) Quantitative analysis of DRP1 levels in hippocampal mitochondrial fractions and AMPK/PGC1α signaling in hippocampal tissues from immunoblots (*n* = 4 mice/group). (f) ATP levels measured in hippocampal tissues of mice (*n* = 5 mice/group). (g) Mitochondrial membrane potential levels in hippocampal tissues of mice (*n* = 5 mice/group). Results are presented as mean ± standard error of the mean (SEM). *p < 0.05; **p < 0.01; ***p < 0.001; ns, not significant.

Mitochondrial dysfunction, characterized by energy deficits and heightened oxidative stress, is associated with POD. The balance between mitochondrial fusion and fission is critical for maintaining mitochondrial function. Following the observed neuroprotective effects of preoperative IF, we further investigated the underlying mechanisms, focusing on mitochondrial function and dynamics. Morphological alterations in mitochondria were examined 24 h post‐anesthesia/surgery using transmission electron microscopy. Compared to the AL‐C group, mitochondria from mice in the AL‐AS group displayed a smaller, more globular, and swollen morphology (Figure 3a). Preoperative IF effectively inhibited the increase in mitochondrial fission observed in the anesthesia/surgery‐challenged hippocampus (Figure 3a).

At the molecular level, we assessed dynamin‐related protein 1 (DRP1), a member of the large GTPase protein family regulating membrane dynamics and morphology. During mitochondrial fission, cytosolic DRP1 assembles into ring‐like structures at mitochondrial constriction sites to promote outer membrane fission. We extracted mitochondria from the hippocampi of all mouse groups using differential centrifugation (Figure 3b) to assess DRP1 expression levels. Anesthesia/surgery induced a significant increase in DRP1 expression compared to controls, and this increase was mitigated by preoperative IF (Figure 3c,e).

To elucidate potential mechanisms underlying downregulation of mitochondrial fission in the IF‐AS group, we assessed expression of AMP‐activated protein kinase (AMPK) and peroxisome proliferator‐activated receptor gamma coactivator 1‐alpha (PGC1α). AMPK mediates cellular energy metabolism and responds to fasting, while PGC1α is the master regulator of mitochondrial quality control. Anesthesia/surgery resulted in significantly decreased levels of phosphorylated AMPK (p‐AMPK) and PGC1α, and these changes were effectively mitigated by preoperative IF (Figure 3d,e). These results suggest that preoperative IF may inhibit anesthesia/surgery‐induced mitochondrial fission in the hippocampus by activating the AMPK–PGC1α pathway.

Further, we assessed mitochondrial function by quantifying ATP levels and mitochondrial membrane potential. Compared to the AL‐C group, mice in the AL‐AS group had significantly decreased ATP levels and mitochondrial membrane potential (Figure 3f,g). Preoperative IF substantially mitigated the decreased ATP production (Figure 3f). While IF also diminished the detrimental effect on mitochondrial membrane potential, this improvement did not reach statistical significance (Figure 3g, *p* = 0.252). Collectively, these findings suggest that preoperative IF preserved mitochondrial ATP production and dynamics in the hippocampus.

### 3.3 IF Reshaped Gut Microbiota and Enhanced Its Tolerance to Anesthesia/Surgery

**FIGURE 4:**
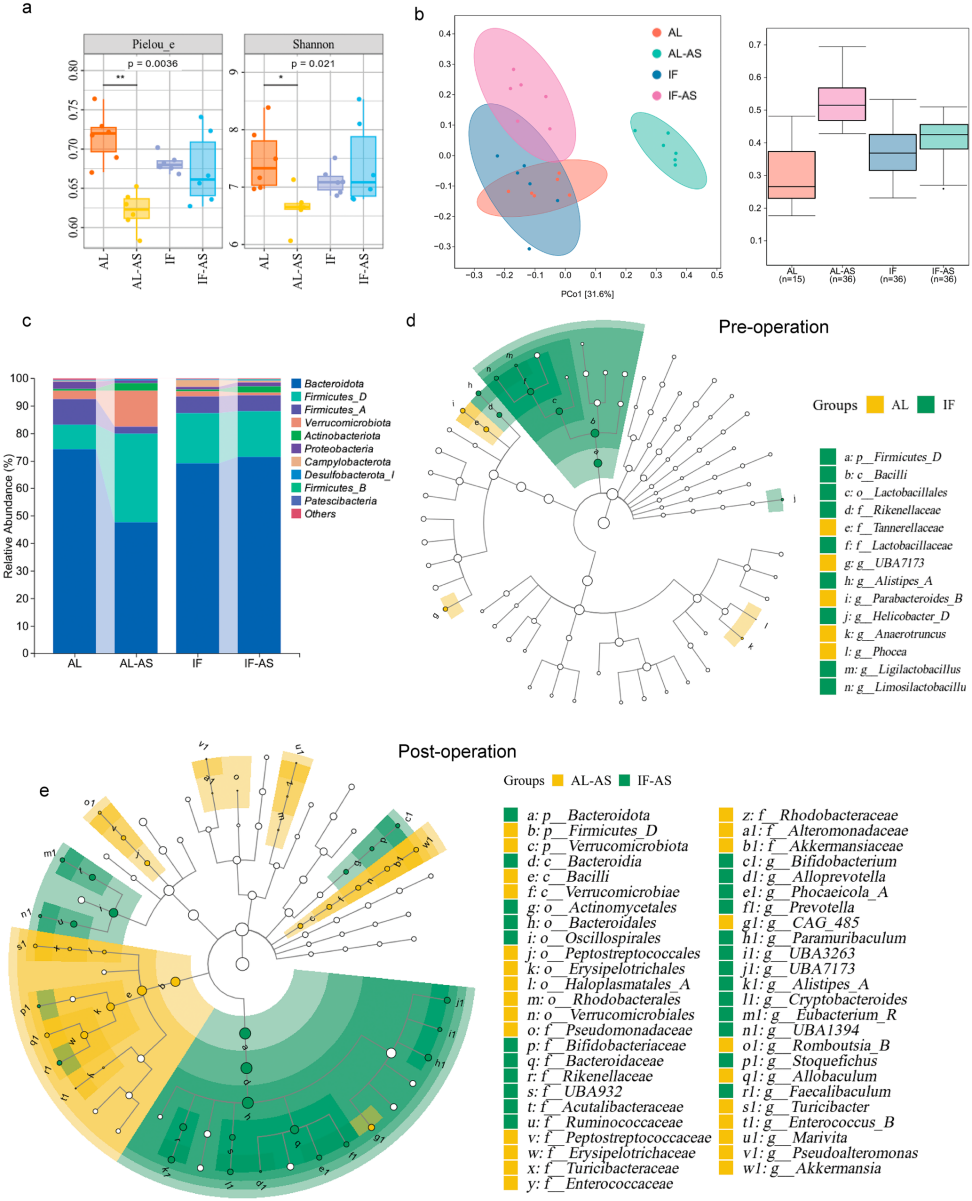
Intermittent fasting reshaped gut microbiota and enhanced its tolerance to anesthesia/surgery (a) Gut microbial diversity assessed by alpha diversity indices for mice in ad libitum (AL) and intermittent fasting (IF) groups, exposed to anesthesia/surgery (AS) or control (C) conditions. In boxplots, center line represents median, and box limits represent upper and lower quartiles (*n* = 6 mice/group). (b) Microbial community structure analyzed using principal coordinates analysis (*n* = 6 mice/group). (c) Stacked bar charts revealed differences at the phylum level (*n* = 6 mice/group) (d) Lefse analyses in taxonomic composition between AL and IF groups (*n* = 6 mice/group). (e) Lefse analyses in taxonomic composition between AL‐AS and IF‐AS groups (*n* = 6 mice/group).

To explore whether the “gut‐brain axis” is involved in the protective effect of IF against postoperative delirium, we collected mouse fecal samples preoperatively (AL, IF) and post‐behavioral assessment (AL‐AS, IF‐AS) and conducted 16S rRNA sequencing. We assessed α‐diversity with the Pielou Index and Shannon Index (Figure 4a) and compared community structure differences using principal coordinate analysis (PCoA) based on weighted UniFrac distances (Figure 4b). Results showed that anesthesia and surgery significantly reduced gut microbiota diversity and disrupted community structure (Figure 4a,b). Preoperative IF effectively alleviated these adverse effects compared to the AL group, indicating IF enhances the gut microbiota's resilience to anesthesia and surgical stress.

Stacked bar charts at the phylum level illustrate perioperative gut microbiota changes in mice across groups (Figure 4c). After anesthesia and surgery, AL mice exhibited significant increases in *Firmicutes*, *Verrucomicrobiota*, and *Actinobacteria*, with concomitant decreases in *Bacteroidetes* and *Proteobacteria*. In contrast, IF mice showed markedly reduced alterations in gut microbiota abundance following the same anesthesia and surgical procedures. LEfSe analysis further distinguished differential microbiota between groups. Preoperatively, compared to AL mice, IF mice had higher levels of *Lactobacillaceae*, *Ligilactobacillu*s, and *Limosilactobacillus* (Figure 4d). Postoperatively, differences between AL‐AS and IF‐AS mice were more evident. Notably, at the genus level, IF‐AS mice were enriched with *Bifidobacterium*, *Prevotella*, and *Alistipes*, whereas the AL‐AS mice showed increased levels of Enterococcus, which is a common opportunistic pathogen observed after surgery [26, 27, 28] (Figure 4a,b).

### 3.4 IF Suppressed the Decline in Fecal Short‐Chain Fatty Acids After Anesthesia /Surgery

**FIGURE 5:**
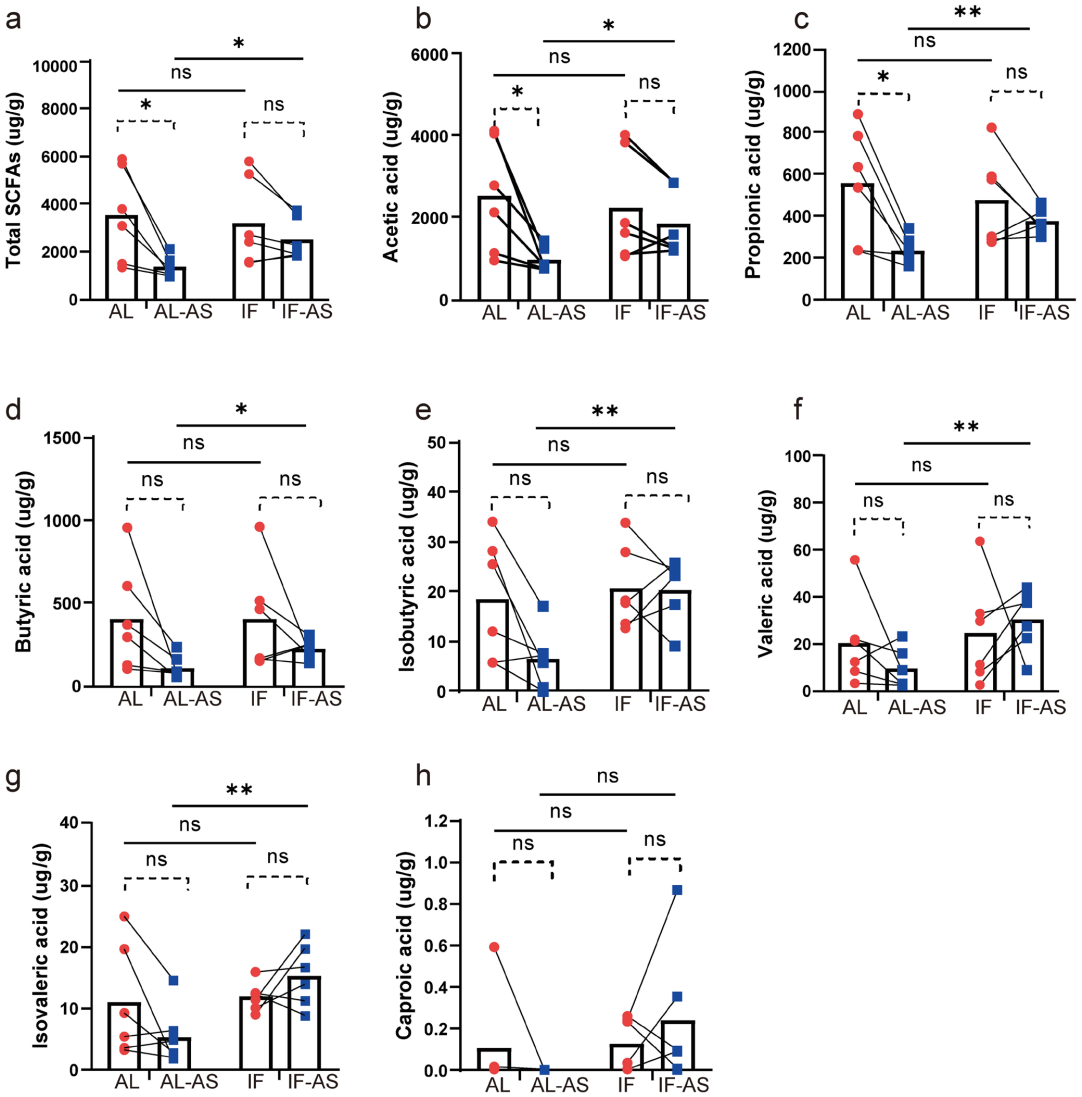
Intermittent fasting suppressed the decline in fecal Short‐Chain Fatty Acids after anesthesia/surgery (*n* = 6 mice/group). Results are presented as the mean and specific numerical values. Dashed lines represent paired t‐tests, while solid lines represent independent samples *t*‐tests. *p < 0.05; **p < 0.01; ns, not significant.

Short‐chain fatty acids (SCFAs) are primary beneficial gut microbiota metabolites. Given the impact of intermittent fasting (IF) on gut microbiota, we assessed the effects of IF on fecal SCFAs in aged mice during anesthesia/surgery (Figure 5). Consistent with our clinical observations [6], anesthesia/surgery reduced total fecal SCFAs of aged mice. Notably, while preoperative IF didn't significantly affect SCFA levels compared to ad libitum (AL) feeding (*p* > 0.05), it effectively suppressed the surgery‐induced decline in SCFAs, particularly acetic acid (*p* < 0.05). Postoperatively, except for caproic acid, IF‐fed mice (IF+AS group) had higher SCFA levels than AL‐fed mice (AL + AS group), indicating that the benefits of IF may stem from preventing the postoperative decrease in gut SCFAs.

**FIGURE 6:**
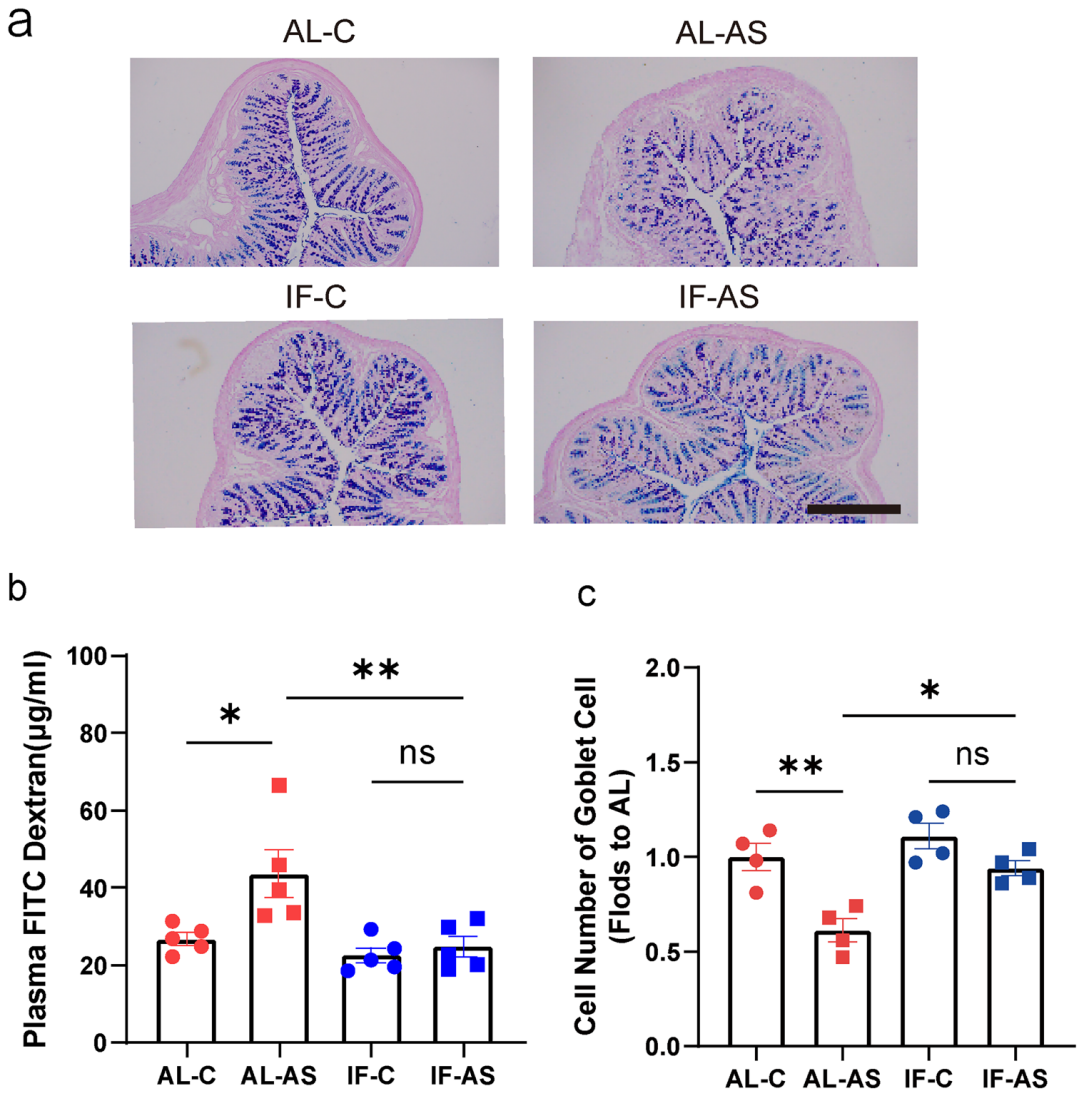
Intermittent fasting mitigates the impact of anesthesia/surgery on intestinal barrier integrity. (a) Levels of FITC‐dextran in mouse plasma 4 h after FITC‐dextran gavage (*n* = 5 mice/group). (b, c) Representative PAS/Alcian blue staining of colon sections (*n* = 4) and corresponding counts of Goblet cells. Results are presented as mean ± standard error of the mean (SEM); *p < 0.05; **p < 0.01; ns, not significant.

Recent studies have linked impaired gut barrier function, commonly referred to as “leaky gut,” with various neurological disorders. Intestinal barrier permeability depends on the integrity of the gut microbiota and is associated with postoperative complications. Therefore, we investigated the effects of preoperative IF on gut barrier integrity during the perioperative period. Before euthanasia, mice were gavaged with FITC‐dextran to assess intestinal permeability. Anesthesia/surgery significantly increased intestinal permeability in aged mice (Figure 6a), alongside a notable decrease in colonic goblet cells post‐surgery (Figure 6b,c) compared to controls. Goblet cells are specialized glandular cells in the intestinal epithelium that secrete mucus, playing a crucial role in maintaining intestinal barrier integrity and function. Preoperative IF effectively reduced the adverse effects of anesthesia/surgery on intestinal permeability (Figure 6a) and colonic goblet cell count (Figure 6b,c).

### 3.5 IF Maintains Hippocampal Mitochondrial Dynamics via the Gut Microbiota

**FIGURE 7:**
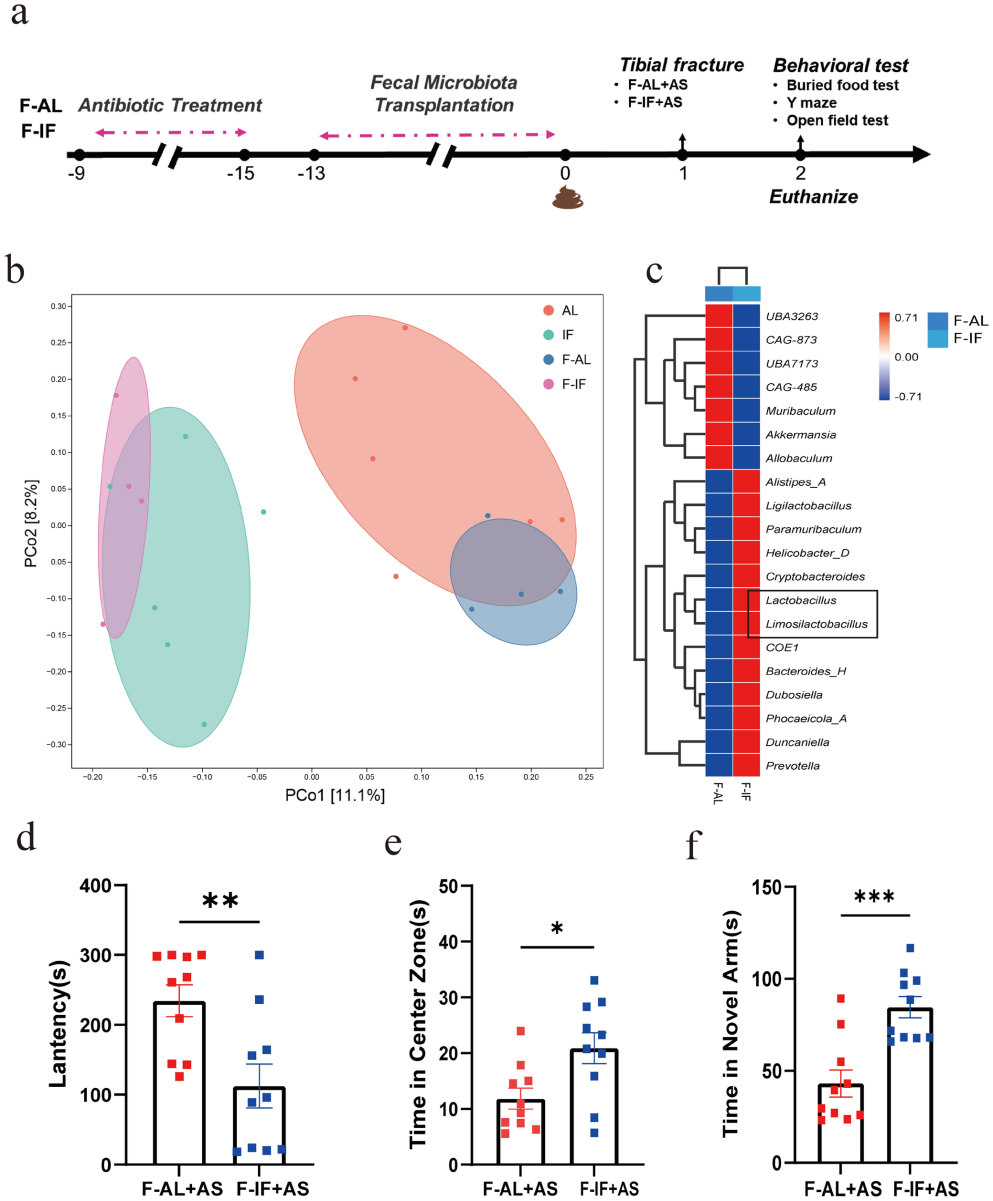
Gut microbiota transplantation from intermittent fasting mice enhances cognitive function post‐surgery. (a) Schematic representation of the fecal microbiota transplantation experiment for mice in ad libitum (AL) or intermittent fasting (IF) groups exposed to anesthesia/surgery (AS). (b) Principal coordinates analysis demonstrates clustering of gut microbiota from fecal microbiota transplant recipient mice with respective donor groups (*n* = 4 ~ 6 mice/group). (c) Heatmap showing relative abundance of the top 20 gut microbiota genera post‐transplantation (*n* = 4 mice/group). (d) Latency of mice to locate food during the buried food test (*n* = 10 mice/group). (e) Time mice spent in the central zone during the open field test (*n* = 10 mice/group). (f) Time mice spent in the novel arm during the Y‐maze test (*n* = 10 mice/group). Results are presented as mean ± standard error of the mean (SEM). *p < 0.05; **p < 0.01; ***p < 0.001; ns, not significant.

To investigate whether alterations of gut microbiota contribute to perioperative neuroprotective effects, we transplanted gut microbiota from mice in the AL and IF groups into 18‐month‐old recipient C57BL/6 female mice (Figure 7a). Principal coordinates analysis demonstrated that the gut microbiota of recipient mice clustered with that of their respective donors, indicating successful transplantation (Figure 7b). To further clarify changes in the composition of gut microbiota after fecal microbiota transplantation, cluster heatmaps were used to demonstrate relative levels of gut microbiota at the genus level (top 20). Results showed that compared with fecal microbiota transplantation from AL mice, levels of *Ligilactobacillus* and *Limosilactobacillus* increased significantly after transplantation from IF mice (Figure 7c).

**FIGURE 8:**
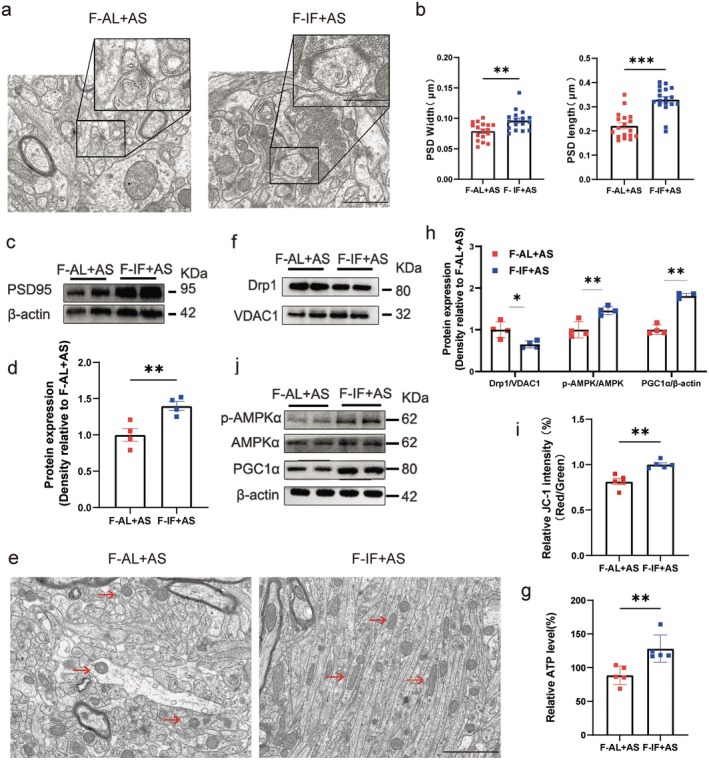
Gut microbiota influence hippocampal synaptic ultrastructure and mitochondrial dynamics post‐transplantation. (a) Representative transmission electron microscopy images of synaptic ultrastructure of mice that received fecal microbiota transplant (F) from ad libitum (AL) or intermittent fasting (IF) group mice exposed to anesthesia/surgery (AS). (b) Measurements of postsynaptic density (PSD) length and width (*n* = 5 slices/group). Scale bar = 1.0 μm (c, d) Western blot images and quantification of PSD95 levels in the hippocampus of mice (*n* = 4 mice/group). (e) Transmission electron microscopy images of mitochondrial morphology of mice. Scale bar = 2.0 μm. Red arrows indicate mitochondria. (f) Quantitative analysis of DRP1 levels in hippocampal mitochondrial fractions and AMPK/PGC1α signaling in hippocampal tissues of mice from immunoblots (*n* = 4 mice/group). (g) Representative western blot images of AMPK/PGC1α signaling pathway in the hippocampus of mice. (h) Representative western blot images of DRP1 levels in hippocampal mitochondrial fractions of mice (*n* = 4 mice/group). (i) ATP levels measured in hippocampal tissues of mice (*n* = 5 mice/group). (j) Mitochondrial membrane potential levels in hippocampal tissues of mice (*n* = 5 mice/group). Results are presented as mean ± standard error of the mean (SEM). *p < 0.05; **p < 0.01; ***p < 0.001; ns, not significant.

After successful transplantation, both groups of mice underwent anesthesia/surgery followed by cognitive assessments. Compared to the F‐AL + AS group (which received gut microbiota from the AL group), F‐IF+AS mice had significantly less latency in finding buried food (Figure 7d) and spent more time in the center area of the open field test (Figure 7e) and in the novel arm of the Y‐maze test (Figure 7f). Ultrastructural evaluation of hippocampal synapses revealed that postsynaptic density length and width were significantly increased in the F‐IF+AS group compared to the F‐AL + AS group (Figure 8a,b). Additionally, the F‐IF+AS group had significantly higher expression of PSD‐95, consistent with the observed postsynaptic density ultrastructural changes (Figure 8c,d).

Based on these results and the well‐documented role of mitochondrial dynamics in influencing mitochondrial function and cognitive processes, we assessed related indicators of mitochondrial dynamics. Using transmission electron microscopy, we found that the F‐AL + AS group had swollen and spherical hippocampal mitochondria, whereas the F‐IF+AS group had mitochondria with relatively normal morphology (Figure 8e). The F‐AL + AS group also had significantly higher DRP1 expression, suggesting that hippocampal mitochondria in this group are predisposed to undergo fission compared to the F‐IF+AS group (Figure 8f,h). Further, AMPK–PGC1α, a key regulator of mitochondrial homeostasis, was activated in the F‐IF+AS group compared to the F‐AL + AS group (Figure 8f,g). Additionally, the F‐IF+AS group had increased ATP and mitochondrial membrane potential levels in hippocampal tissue compared to the F‐AL + AS group (Figure 8i,j). These findings from fecal microbiota transplantation indicate that gut microbiota plays a crucial role in regulating the neuroprotective effects of IF, maintaining homeostasis of mitochondrial dynamics.

### 3.6 SCFAs Can Exert Neuroprotective Effects During the Perioperative Period

**FIGURE 9:**
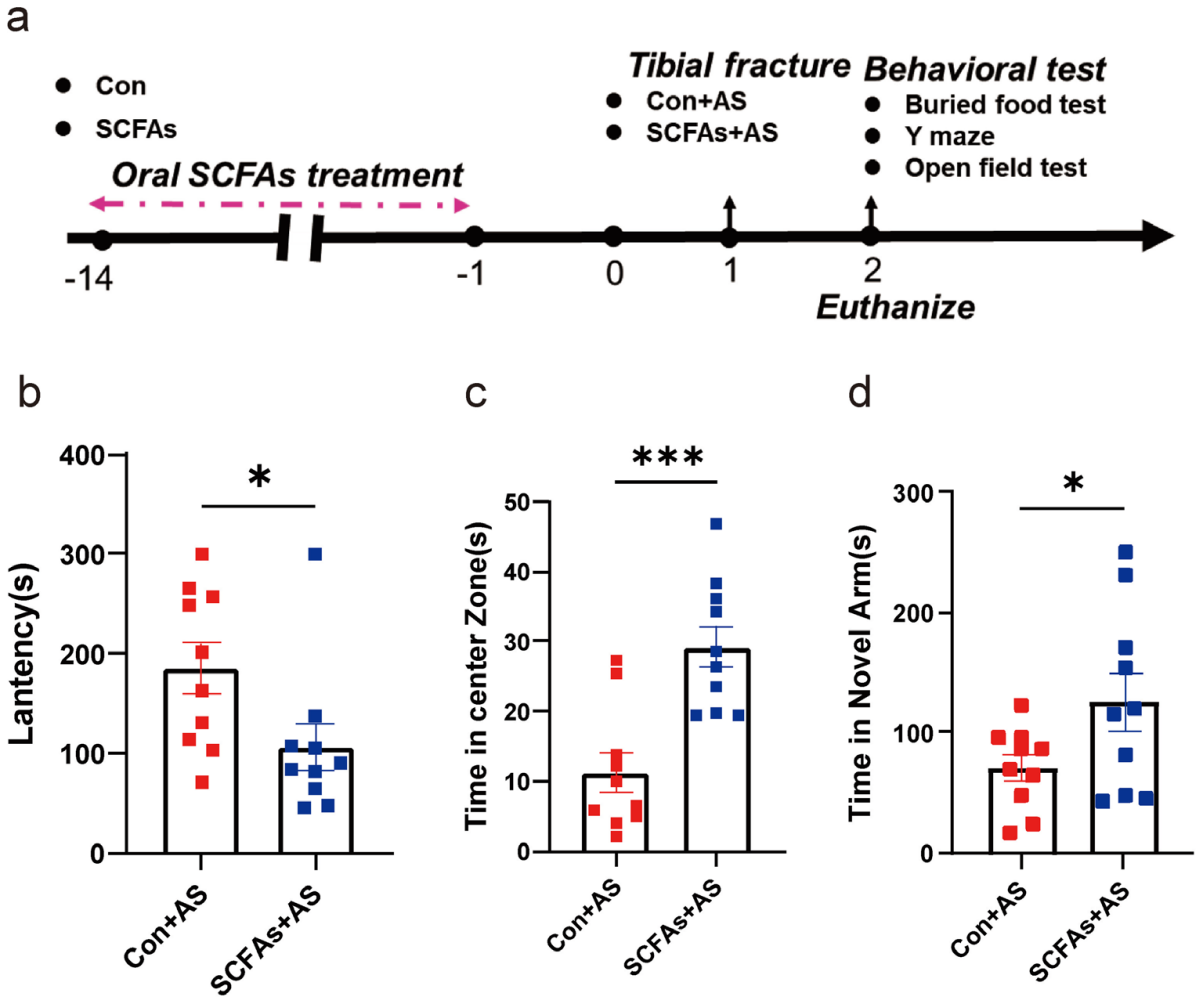
Short‐chain fatty acid supplementation alleviates anesthesia/surgery‐induced behavioral deficits. (a) Schematic representation of the experimental design for short‐chain fatty acids (SCFAs) supplementation. Mice received drinking water supplemented with a cocktail of SCFAs (acetate, propionate, and butyric acid) or plain drinking water (Con) for 14 days. subsequently, all groups were exposed to anesthesia/surgery (AS). (b) Latency to locate food during the buried food test conducted post‐surgery (*n* = 10 mice/group). (c) Time spent in the central zone during the open field test post‐surgery (*n* = 10 mice/group). (d) Time spent exploring the novel arm during the Y‐maze test post‐surgery (*n* = 10 mice/group). Results are presented as mean ± standard error of the mean (SEM). *p < 0.05; ***p < 0.001; ns, not significant.

**FIGURE 10:**
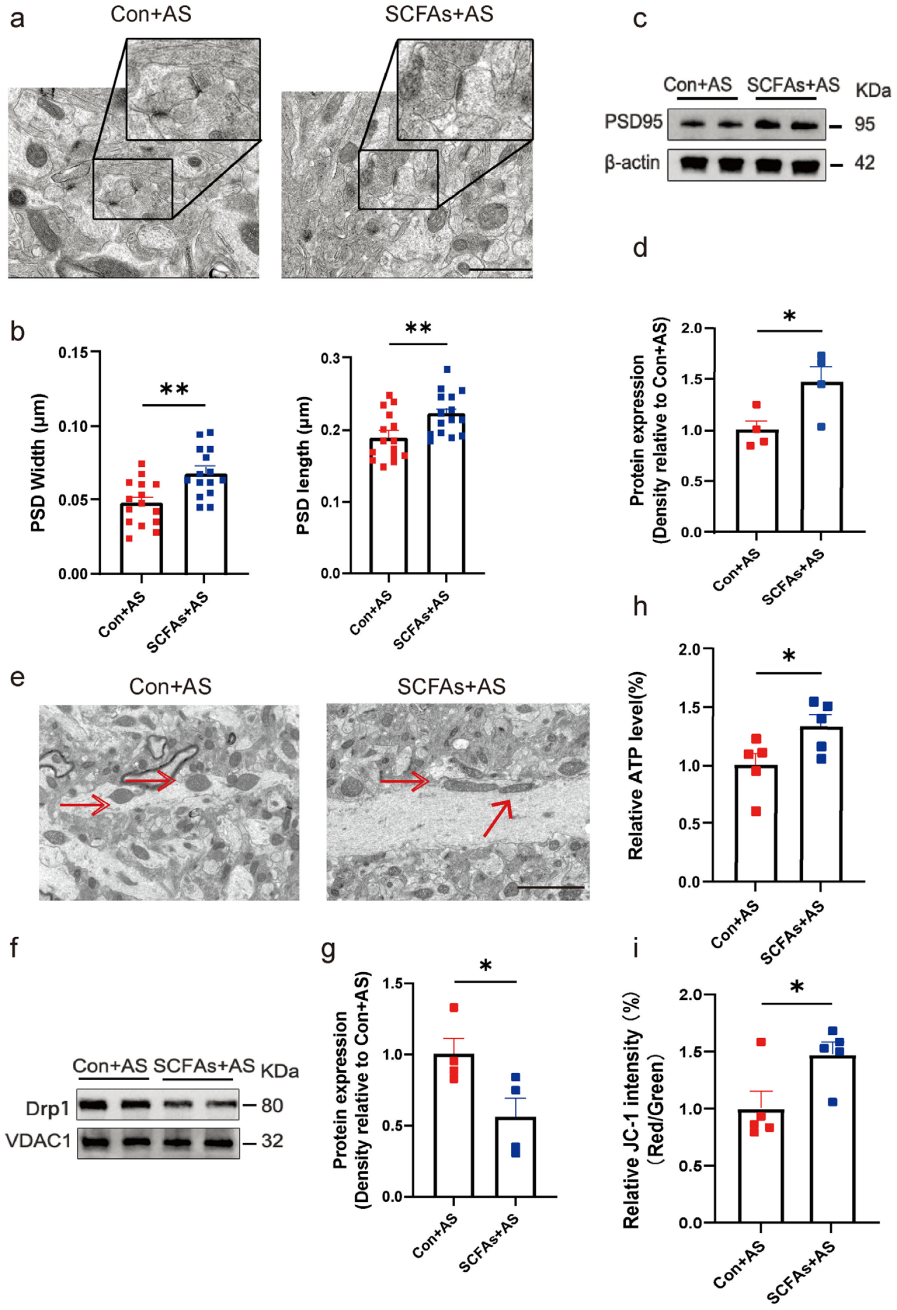
Short‐chain fatty acid supplementation protects hippocampal synaptic integrity and mitochondrial function post‐anesthesia/surgery. (a) Representative transmission electron microscopy images of synaptic ultrastructure of mice that SCFA‐supplemented mice (SCFAs+AS) compared to controls (Con+AS) post‐surgery. Scale bar = 1.0 μm (b) Measurements of postsynaptic density (PSD) length and width (*n* = 5 slices/group). (c, d) Western blot images and quantification of PSD95 levels in the hippocampus of mice (*n* = 4 mice/group). (e) Transmission electron microscopy images of mitochondrial morphology of mice. Scale bar = 2.0 μm. Red arrows indicate mitochondria. (f, g) Western blot images and quantification of DRP1 levels in hippocampal mitochondrial fractions (*n* = 4 mice/group). (h) ATP levels measured in hippocampal tissues of mice (*n* = 5 mice/group). (i) Mitochondrial membrane potential levels in hippocampal tissues of mice (*n* = 5 mice/group). Results are presented as mean ± standard error of the mean (SEM). *p < 0.05; **p < 0.01; ns, not significant.

To experimentally demonstrate whether IF‐enriched SCFAs could mimic the protective effects of IF on aged mice undergoing anesthesia/surgery, we administered a cocktail of SCFAs (acetate 67.5 mM, propionate 40 mM, and butyric acid 25 mM) dissolved in the drinking water to aged mice for 14 days (Figure 9a). Compared to the control mice (Con+AS), the SCFAs‐treated aged mice (SCFAs+AS) displayed better natural behavior during the open field test and the buried food test, as well as better cognitive ability in the Y‐maze test (Figure 9b–d). Consistent with these behavioral analyses, SCFAs administration also conferred significant neuroprotection at the cellular level. Ultrastructural evaluation revealed that the SCFAs+AS group had enhanced hippocampal synaptic integrity, characterized by significantly increased postsynaptic density (PSD) length and width (Figure 10a,b) and higher expression of the key scaffolding protein PSD‐95 compared to the Con+AS group (Figure 10c,d). At the mitochondrial level, SCFAs supplementation similarly reversed the adverse effects of surgery. Compared to the Con+AS group, the SCFAs+AS group exhibited more normal hippocampal mitochondrial morphology (Figure 10e), significantly lower expression of the key fission protein DRP1 (Figure 10f,g), and significantly increased ATP levels and mitochondrial membrane potential (Figure 10h,i). These findings indicate that short‐chain fatty acids, as key gut microbiota metabolites, play a crucial role in mediating these neuroprotective effects.

## 4 Discussion

Our results demonstrate that preoperative IF can mitigate anesthesia/surgery‐induced delirium‐like behaviors in aged mice. The gut microbiota following IF intervention helps mitigate the postoperative decline in short‐chain fatty acid levels, alleviate anesthesia/surgery‐induced mitochondrial fission in the hippocampus, and maintain hippocampal mitochondrial function during the perioperative period.

In this study, we utilized aged mice undergoing tibial fracture internal fixation under sevoflurane anesthesia to model postoperative delirium, a widely recognized approach [29, 30]. Common methods for creating postoperative delirium models in mice also include laparotomy or thoracotomy in older mice [31, 32]. In comparison to these methods, the model used in our study is relatively straightforward and easy to control. Moreover, it avoids direct interference with the intestine, allowing for a more objective assessment of the role of gut microbiota in postoperative delirium.

Aging is recognized as one of the most critical independent risk factors for POD. Our study confirmed that anesthesia and surgery induce substantial gut microbiota disturbances in aged mice, a phenomenon less observed in young mice [4, 33]. As aging progresses, the gut microbiota undergoes distinct alterations, including reduced microbial diversity and increased instability, rendering aged individuals more susceptible to dysbiosis following surgical stress [20]. Consistent with these age‐dependent characteristics, our aged mice exhibited a significant reduction in gut microbiota diversity following anesthesia and surgery.

Diet is a crucial intervention for modulating the composition and function of gut microbiota. IF is characterized by alternating periods of fasting and unrestricted eating, primarily focusing on meal timing rather than dietary composition [8, 9]. Our findings indicate that IF significantly increased the abundance of *Lactobacillaceae*, particularly *Ligilactobacillus* and *Limosilactobacillus*, which are known SCFA‐producing bacteria. Notably, these genera were reclassified from the traditional *Lactobacillus* genus in 2020. Because the host genome lacks the polysaccharide lyases necessary to digest many complex carbohydrates, these substrates are instead fermented in the colon by gut bacteria possessing carbohydrate‐active enzymes (CAZymes)—such as *Lactobacillus* and *Bifidobacterium*—to produce SCFAs [34, 35]. During periods of food scarcity, microbial taxa that can readily adapt to shifts in energy sources—such as *Lactobacillaceae*, which possess a large repertoire of CAZyme‐encoding genes—exhibit a marked increase in abundance [36, 37]. Despite variations in animal strains, housing conditions, and fasting protocols, multiple studies have demonstrated that IF consistently enhances the abundance of *Lactobacillaceae* [21, 36, 38]. While previous research had not explicitly linked the beneficial effects of preoperative IF to gut microbiota alterations, existing animal studies have shown that preoperative administration of *Lactobacillus* can mitigate postoperative delirium‐like behaviors following anesthesia and surgery. Crucially, through fecal microbiota transplantation, we demonstrated that transferring gut microbiota from mice subjected to IF reduced anesthesia/surgery‐induced delirium‐like behaviors in recipient mice. This provides direct evidence that the beneficial effects of IF are mediated by the gut microbiota.

Furthermore, our results highlight the critical role of mitochondrial dynamics in perioperative neurocognitive disorders. Nutrient‐rich conditions tend to promote mitochondrial fission, while starvation encourages fusion to maximize energy efficiency [11]. We found that 30‐day preoperative IF mitigated anesthesia‐induced mitochondrial fission in the hippocampus. Interestingly, we observed no significant differences in mitochondrial dynamic indicators between the baseline control (AL‐C) and IF control (IF‐C) groups; this is likely due to the *ad libitum* re‐feeding period both groups experienced prior to tissue collection. The disruption of mitochondrial dynamics in neurons, microglia, and astrocytes by anesthesia/surgery can exacerbate neuroinflammation and lead to neuronal apoptosis [37, 39]. Our data suggest IF helps maintain mitochondrial homeostasis against these insults.

SCFAs act as key intermediate transmitters in the gut‐brain axis. Our SCFA rescue experiment confirmed their neuroprotective role, aligning with clinical observations linking lower postoperative SCFA levels to greater delirium severity [6]. The mechanisms by which SCFAs exert these neuroprotective effects are likely multimodal, involving direct humoral, neural, and endocrine pathways. Humorally, SCFAs can cross the blood–brain barrier to exert direct neuroprotection on neuronal cell function via monocarboxylate transporters and specific receptors (e.g., FFAR2, FFAR3) expressed on neural cells [40]. Recent studies suggest SCFAs directly influence neuronal mitochondrial function; for instance, propionate maintains neuronal mitochondrial dynamics and autophagy homeostasis, thereby improving cognitive function, while butyrate can alleviate suppression of neuronal mitophagy [41, 42]. Neurally, increased levels of metabolites like SCFAs may act as signaling molecules to activate vagal afferent fibers within the gut [43]. These signals are subsequently transmitted to central nervous system structures such as the nucleus tractus solitarius (NTS), further influencing cerebral function. Supporting this vagal mechanism, recent research indicates that *Roseburia intestinalis* ‐derived butyrate alleviates neuropathic pain by stimulating the vagus nerve, and notably, the cognitive protective effects of SCFAs in Alzheimer's disease mice are attenuated following vagotomy [44, 45]. While our rescue experiment confirms the importance of SCFAs, it does not distinguish whether their primary effects are mediated through systemic circulation or via the vagus nerve pathway.

This study has several limitations that warrant acknowledgement. First, given the broad systemic benefits of IF, isolating a single specific molecular target for mechanistic studies via pharmacological antagonists or knockout models remains challenging. Second, aged female mice were used exclusively in this study. This choice reflects reports that females exhibit greater vulnerability to anesthesia‐induced cognitive impairment [19] and aligns with clinical findings of increased POD incidence among elderly women [46]; therefore, our results may not be directly generalizable to males. Future studies incorporating male animals are essential to explore potential sex‐based differences. Third, our analysis focused primarily on the hippocampus; other cognition‐related brain regions vulnerable to anesthetic effects, such as the prefrontal cortex (PFC), also merit investigation. Finally, we did not perform a vagotomy experiment to definitively confirm the specific role of the vagus nerve in mediating the observed gut‐brain communication.

In conclusion, our study demonstrates that preoperative intermittent fasting confers neuroprotection against anesthesia/surgery‐induced delirium‐like behaviors in aged mice. This effect is mediated by the remodeling of gut microbiota and the preservation of SCFAs levels, which in turn maintain hippocampal mitochondrial dynamics and function. These findings suggest that preoperative dietary interventions could serve as a viable strategy for preventing postoperative delirium.

## Funding

The research was supported by Hebei Medical University Postdoctoral Fund, the Postdoctoral Research Support Program for Clinical Medicine of Hebei Medical University (PD2025007), and the S&T Program of Hebei (No. H2022206586).

## Ethics Statement

All animal experiments were approved by the Animal Ethics Committee at The Second Hospital of Hebei Medical University (Approval Letter No. 2024‐AE285).

## Conflicts of Interest

The authors declare no conflicts of interest.

## Data Availability

The data presented in the study are deposited in the NCBI (https://www.ncbi.nlm.nih.gov/) repository. Accession number is PRJNA1295579.
